# Electroacupuncture promotes the survival and synaptic plasticity of hippocampal neurons and improvement of sleep deprivation‐induced spatial memory impairment

**DOI:** 10.1111/cns.13722

**Published:** 2021-10-08

**Authors:** Wenya Pei, Fanqi Meng, Qingwen Deng, Baobao Zhang, Yuan Gu, Boyu Jiao, Haoyu Xu, Jiuqing Tan, Xin Zhou, Zhiling Li, Guanheng He, Jingwen Ruan, Ying Ding

**Affiliations:** ^1^ Department of Acupuncture The First Affiliated Hospital Sun Yat‐sen University Guangzhou China; ^2^ Department of Histology and Embryology Zhongshan School of Medicine Sun Yat‐sen University Guangzhou China; ^3^ Guangzhou Provincial Hospital of Chinese Medicine Guangzhou China; ^4^ Guangzhou University of Traditional Chinese Medicine Guangzhou China; ^5^ Department of Traditional Chinese Medicine The Seventh Affiliated Hospital Sun Yat‐sen University Shenzhen China

**Keywords:** brain‐derived neurotrophic factor, electroacupuncture, hippocampal neuron, memory impairment, sleep deprivation, synaptic plasticity

## Abstract

**Aims:**

This study aimed to investigate whether electroacupuncture (EA) promotes the survival and synaptic plasticity of hippocampal neurons by activating brain‐derived neurotrophic factor (BDNF)/tyrosine receptor kinase (TrkB)/extracellular signal‐regulated kinase (Erk) signaling, thereby improving spatial memory deficits in rats under SD.

**Methods:**

In vivo, Morris water maze (MWM) was used to detect the effect of EA on learning and memory, at the same time Western blotting (WB), immunofluorescence (IF), and transmission electron microscopy (TEM) were used to explore the plasticity of hippocampal neurons and synapses, and the expression of BDNF/TrkB/Erk signaling. In vitro, cultured hippocampal neurons were treated with exogenous BDNF and the TrkB inhibitor K252a to confirm the relationship between BDNF/TrkB/Erk signaling and synaptic plasticity.

**Results:**

Our results showed that EA mitigated the loss of hippocampal neurons and synapses, stimulated hippocampal neurogenesis, and improved learning and memory of rats under SD accompanied by upregulation of BDNF and increased phosphorylation of TrkB and Erk. In cultured hippocampal neurons, exogenous BDNF enhanced the expression of synaptic proteins, the frequency of the postsynaptic currents, and the phosphorylation of TrkB and Erk; these effects were reversed by treatment with K252a.

**Conclusions:**

Electroacupuncture alleviates SD‐induced spatial memory impairment by promoting hippocampal neurogenesis and synaptic plasticity via activation of BDNF/TrkB/Erk signaling, which provided evidence for EA as a therapeutic strategy for countering the adverse effects of SD on cognition.

## Graphical abstract

Electroacupuncture may ameliorate spatial memory impairment during sleep deprivation through improving hippocampal neuron survival, neurogenesis and synaptic plasticity via regulating BDNF/TrkB/ERK signaling

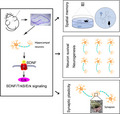

## 1 INTRODUCTION

Sleep deprivation (SD) affects brain function, especially cognition.^1^ Evidence from animal and human studies indicates that SD directly affects memory processes in the hippocampus.^2^, ^3^ SD is associated with reduced cell proliferation and neuron survival, and it inhibits the differentiation of neural progenitor cells into neurons.^4^, ^5^ Sleep and sleep loss influence synaptic efficacy, and the plasticity of neurons and synapses is critical for brain function and cognitive performance.^6^, ^7^, ^8^ SD was shown to induce changes in synapse structure and decrease synaptic plasticity, particularly in the hippocampus.^9^

Brain‐derived neurotrophic factor (BDNF) plays a critical role in learning and memory by regulating neuronal survival and differentiation and modulating and synaptic function.^10^, ^11^, ^12^ Acute SD in patients with primary and secondary sleep disorders was associated with decreased serum BDNF levels.^13^, ^14^ In cultured hippocampal neurons and slices, application of exogenous BDNF activated tyrosine receptor kinase (Trk)B signaling and led to the recruitment of downstream signaling molecules such as mitogen‐activated protein kinase (MAPK)/extracellular signal‐regulated kinase (Erk).^15^ In vivo studies demonstrated that BDNF induced long‐lasting synaptic strengthening in the rodent hippocampus via MAPK/Erk activation.^16^, ^17^, ^18^ Meanwhile, SD altered BDNF/TrkB/Erk signaling to reduce synaptic efficacy in the hippocampus.^19^, ^20^

Commonly used measures to counter the physiologic effects of SD such as adenosine and caffeine have been shown to affect neuronal plasticity,^21^ but they do not mitigate the negative impact of severe sleep loss on higher‐order cognitive functions. Electroacupuncture (EA) has demonstrated efficacy and safety in the treatment of sleep disorders and was shown to increase BDNF level in the brain while improving cognitive function.^22^, ^23^, ^24^, ^25^ We previously reported that EA improved sleep quality and reduced the incidence of adverse events associated with sleep disorders.^26^, ^27^ Moreover, EA treatment prevented memory impairment and altered neurotransmitter levels under acute SD.^28^ Previous studies suggested that acupuncture protects against cognitive impairment by activating multiple neuroprotective molecules.^29^, ^30^ However, the mechanism by which EA improves cognitive impairment under SD is unknown.

In this study, we investigated the mechanistic basis for the effect of EA on cognition under SD. The results of in vitro and in vivo experiments showed that EA promoted the survival and synaptic plasticity of hippocampal neurons by activating BDNF/TrkB/Erk signaling, leading to an improvement in spatial memory under SD. Thus, EA induces molecular and structural changes in the brain that mitigate the negative effects of sleep loss on cognition.

## 2 METHODS

### 2.1 Animals

Adult male Sprague Dawley rats (220–250 g) were provided by the Experimental Animal Center of Sun Yat‐sen University. The animals were housed under standard environmental conditions at 22°C on a 12:12‐h light/dark cycle. After 7 days of adaptation to the laboratory environment, the animals were randomly divided into three groups: Sham, SD, and EA + SD. The investigators performing behavioral testing were blinded to the rats’ group assignment. Experiments involving the rats were approved by the Animal Care and Use Committee of Sun Yat‐sen University (approval no. 2018000679) and were conducted in accordance with the ARRIVE guidelines (Animal Research: Reporting of In Vivo Experiments) guidelines for Animal Research.^31^

### 2.2 Induction of SD

We used the modified multiple platform method (MMPM) to induce SD in rats.^32^ The device used for SD consisted of six cylindrical platforms with a height of 60 mm and diameter of 50 mm, spaced 45 mm apart and fixed in a plastic cage (105 × 55 × 27 cm). The cage was filled with water up to about 1 cm below the surface of platforms. Rats in the SD and EA + SD groups were placed in separate cages to prevent social interaction. Food and water were regularly provided through a grid at the top of the cage. The rats were placed on and allowed to adapt to the platform, and those in the SD and EA + SD groups were continuously subjected to SD 24 h a day for 4 days. Rats in the Sham group which were placed on the grid of the same cage without adding water were allowed to sleep during this time, and those in the other two groups were allowed to sleep when the water in the cage was changed.

### 2.3 EA treatment

**FIGURE 1:**
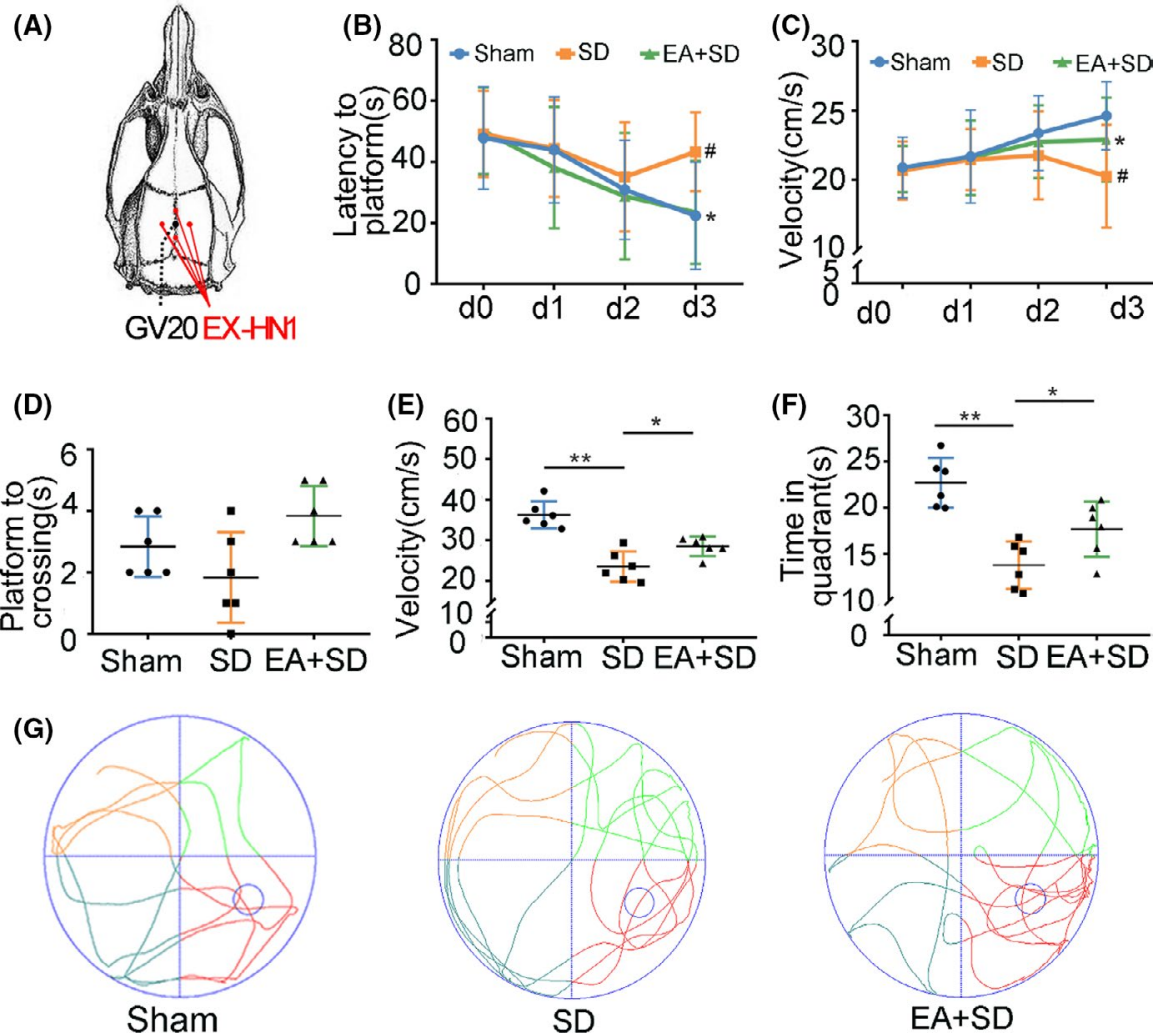
EA alleviates spatial memory impairment induced by SD. (A) The location of acupoints (EX‐HN1) in EA treatment. (B, C) Latency to find the platform (B) and swimming speed (C) in the MWM test. (*n* = 6/group, data were presented as the mean ± SEM and analyzed by nonparametric test (Kruskal‐Wallis test), *p < 0.05 indicates significant difference from the SD group. #*p* < 0.05 indicates significant difference from the EA + SD group) (D–F) Number of platform crossings (D), swimming speed (E), and time in the target quadrant (F) in the probe test. (*n* = 6/group, data were presented as the mean ± SEM and analyzed by least significant difference test (LSD),* *p* < 0.05, ** *p* < 0.01)(G) Trace plot from the probe test without a hidden platform

Electroacupuncture was performed at two sets of Si Shencong acupoints (EX‐HN1), the corresponding sites on the rat are situated on 1 mm posterior, anterior, and lateral to GV20 (Figure 1A, Figure S1E) according to the principle of comparative anatomy. The low‐frequency electronic pulse therapy instrument (G6805‐2A, Shanghai Huayi Medical Instrument Co.) was used among two sets of acupoints. EA was performed daily for 4 days in 20‐min sessions and the current intensity cycle between the acupoint pair was ∼3 μA.^33^, ^34^

### 2.4 Evaluation of neurogenesis

Proliferating cells including ependymal cells, stem cells, and newborn hippocampal neurons were labeled with 5‐bromo‐2′‐deoxyuridine (BrdU; Cat. No B5002, Sigma‐Aldrich). The compound was dissolved in sterile saline at 10 mg/ml and administered by intraperitoneal injection at 50 mg/kg daily for four consecutive days; 2 h after the last injection, rats were anesthetized and perfused with 4% paraformaldehyde in phosphate‐buffered saline (PBS).

### 2.5 Spatial memory test

The Morris water maze (MWM) consisted of a constant temperature swimming pool and data collection and analysis system. The pool was divided into four equal quadrants. We first evaluated visual acuity, motivation to swim, and exercise ability by placing the rats in the pool and allowing them to find the visible platform. Within 24 h after the end of the assessment, the animals underwent 4 days of maze training. In trials lasting 60 s, the time required to find the hidden platform (latency) and swimming course (distance traveled) was recorded. The platform was removed for the probe test within 24 h after the last training trial. The rats were placed in different maze quadrants in a randomized order across trials.

### 2.6 Nissl staining

Brain sections were cut at a thickness of 20 μm and incubated with Nissl staining solution for 20 min at 37°C. Every fifth section throughout the entire rostral–caudal extent of the hippocampus was examined under a fluorescence microscope (Olympus) to quantify the numbers of neurons in different subregions of the hippocampus (CA1, CA2, CA3, dentate gyrus [DG]1, and DG2; 0.56 × 0.37 mm). Cells with darkly stained shrunken cell bodies or a fragmented nuclei were excluded from the analysis.^35^ Counting was performed by an experimenter blinded to the study objectives.

### 2.7 Western blotting

Rats were sacrificed, and the brain was rapidly dissected on ice. The hippocampus was isolated and homogenized in cold radioimmunoprecipitation assay buffer (Beyotime) containing protease inhibitor cocktail and centrifuged at 18,800 *g* for 15 min at 4°C. Protein concentration was determined with the BCA Protein Assay Kit (Invitrogen), and equal amounts were resolved by sodium dodecyl sulfate–polyacrylamide gel electrophoresis on a 10% gel and transferred to a nitrocellulose membrane that was blocked at room temperature for 1 h, then incubated overnight at 4°C with primary antibodies followed by horseradish peroxidase‐conjugated secondary antibodies for 1 h at room temperature. Protein bands were visualized using the Image Quant LAS 4000 mini detection system (GE Healthcare), and immunoreactivity was quantified using ImageJ software (National Institutes of Health) and normalized to that of the loading control β‐actin (or total protein in the analysis of phosphorylated protein levels).

### 2.8 Immunofluorescence analysis

The brain was sectioned at a thickness of 25 μm on a cryostat for immunofluorescence labeling. The sections were blocked with 10% goat serum for 30 min at 37°C and then incubated overnight at 4°C with primary antibodies. After rinsing in PBS, the sections were incubated with secondary antibodies, and Hoechst33342 was used to stain the nuclei. The sections were examined with a fluorescence microscope (Leica) and laser confocal microscope (LSM800; Zeiss).

To assess cell proliferation, brain tissue sections were incubated in 2 N hydrochloric acid at 37°C for 30 min and washed in 0.1 M sodium borate (pH 8.5) in PBS. They were then incubated overnight at 4°C with primary antibodies against neuronal nuclei (NeuN), doublecortin (DCX), and BrdU^36^, followed by secondary antibodies. The sections were observed with a laser confocal microscope, and BrdU‐labeled cells were quantified. A list of the antibodies used in this experiment is provided in Table S1.

### 2.9 Transmission electron microscopy

The hippocampus was cut into 1 × 1 × 1 mm pieces that were fixed with 2.5% glutaraldehyde, 4% paraformaldehyde, and 1% picric acid. The pieces were stained with osmic acid for 2 h and embedded overnight in Epon812, then polymerized at 60°C for 48 h. Semithin sections (2 µm) were cut, stained with toluidine blue, and mounted with neutral balsam. For analysis by transmission electron microscopy (TEM) (CM10, Philips), ultrathin 100‐nm sections were cut and stained with lead citrate and uranyl acetate. Briefly, there were five samples in each group, and the 5^th^ ultrathin serial section of each sample was selected and photographed. A total of five field (0.31 mm × 0.31 mm) images were randomly captured from the selected ultrathin‐section; in other words, synapses were counted and analyzed in 25 unit areas (0.31 mm × 0.31 mm) per group. ImageJ software was used to analyze synapse structure as previously described.^37^, ^38^

### 2.10 Primary hippocampal neuron culture

The hippocampus of newborn rats was isolated and dissociated into a single‐cell suspension. The cells were resuspended in Dulbecco’s modified Eagle’s medium with 10% fetal bovine serum and seeded at a density of 4.0–6.0 × 10^5^ cells/ml in 24‐well or six‐well plates coated with poly‐d‐lysine (0.1 mg/ml) in neurobasal medium supplemented with 2% B27, 1% N_2_, 1% GlutaMax, and 1% penicillin/streptomycin. The neurons were cultured for 10 days before they were used in experiments.

Primary hippocampal neurons were randomly divided into control, BDNF, K252a, and K252a + BDNF groups. The BDNF and K252a groups were treated with BDNF (80 ng/ml) or the TrkB kinase inhibitor K252a (50 μM) (both from Sigma‐Aldrich), respectively, for 24 h. In the K252a + BDNF group, cultures were pretreated with K252a for 12 h followed by BDNF for 12 h.

### 2.11 Whole‐cell patch clamp

Hippocampus neurons were transferred into a recording chamber. Artificial cerebrospinal fluid bubbled with 95% O_2_ and 5% CO_2_ was perfused into the recording chamber using a peristaltic pump (HEKA Inc.) with a constant speed 3–4 of ml/min. Cells were held in the current‐clamp mode, and their firing properties were assessed by delivering 600‐ms depolarizing current steps. To record miniature excitatory postsynaptic currents (mEPSCs), cells were held on voltage‐clamp mode and patch pipettes (2–4 MΩ) were filled with the internal solution. Cells were held at −70 mV to record mEPSCs. All data were filtered at 3 kHz and digitized at 10 kHz using Igor Pro (Wave Metrics).

### 2.12 Enzyme‐linked immunosorbent assay (ELISA)

The culture medium of primary hippocampal neurons treated with BDNF or K252a was replaced with regular medium. The culture supernatant was collected 24 h later, centrifuged (4°C, 18800 *g*, 15 min), and stored at −80°C until use. BDNF levels in the supernatant were measured using a BDNF ELISA kit (Boster Bio; cat. no. EK0308) according to the manufacturer’s instructions.

### 2.13 Statistical analysis

SPSS v25.0 software (IBM Corp.) was used to perform statistical analysis. Data are expressed as mean ± standard deviation and analyzed using one‐way analysis of variance (ANOVA). If the data were normally distributed, the least significant difference test (LSD) was applied; otherwise, nonparametric Kruskal‐Wallis tests were used. In all tests, *p* < 0.05 was considered statistically significant.

## 3 RESULTS

### 3.1 EA alleviates spatial memory impairment induced by SD

Considering the survival rate and the role of sleep recovery, the modified multiple platform method (MMPM) was used to deprive sleep for 4 days (Figure S1C,D). During SD, we observed a change in the numbers of sleep episodes (Figure S1B). In the MWM training phase, learning occurred gradually across repeated trials (Figure S1A). The latency to reach the platform decreased in all groups, but the SD group required more time to find the platform than the other groups (Figure 1B). Swimming speed increased in the sham and EA + SD groups but decreased in the SD group (Figure 1C). On the third day, both latency to locate the platform and swimming speed differed significantly between the EA + SD and SD groups (Figure 1B, C).

The track plots in the probe test showed that the SD group spent less time in the target quadrant than the other groups (Figure 1D,F,G), but there was no difference in the number of platform crossings among the three groups (Figure 1D). The EA group also had a shorter swimming time than the SD group (Figure 1E,F). These data indicate that EA alleviates SD‐induced learning and memory impairment.

### 3.2 EA enhances neuron survival and neurogenesis under SD

**FIGURE 2:**
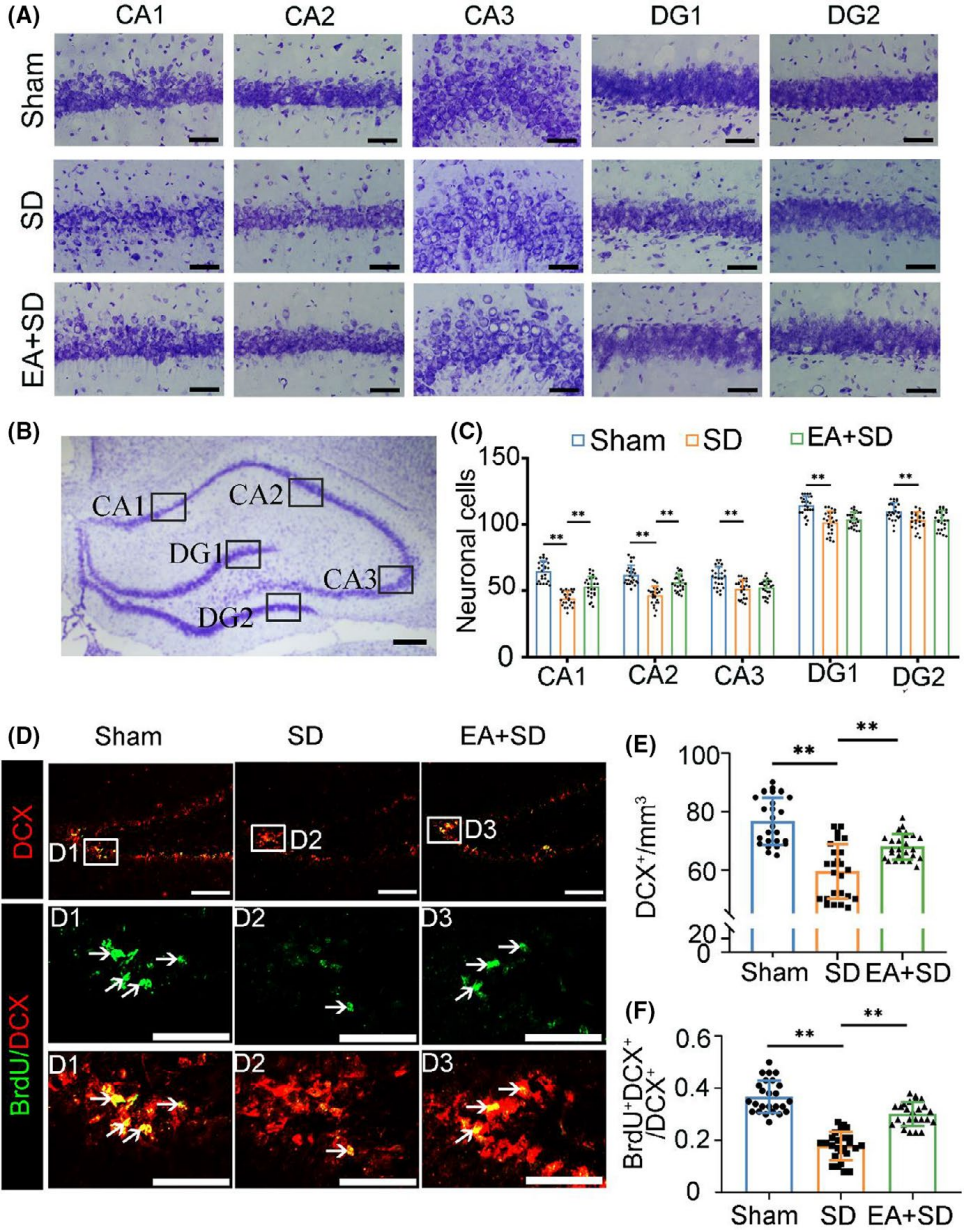
EA enhances neuron survival in the hippocampus and preserves neurogenesis under SD. (A) Representative images of Nissl staining in different regions of the hippocampus. Scale bar, 100 µm. (B) Schematic illustration of different regions of the hippocampus. Scale bar, 50 µm. (C) Quantification of neurons in the hippocampus. (*n* = 5/group, data were presented as the mean ± SEM and analyzed by least significant difference test (LSD), *p < 0.05, **p < 0.01). (D) Colocalization of DCX (red) and BrdU (green) in the hippocampus. Scale bar, 50 µm. Boxed areas show higher magnification views. (E) Quantification of DCX^+^ cells in the DG region of the hippocampus. (*n* = 5/group, data were presented as the mean ± SEM and analyzed by least significant difference test (LSD), *p < 0.05, **p < 0.01). (F) Percentage of BrdU + DCX + /DCX + cells. (*n* = 5/group, data were presented as the mean ± SEM and analyzed by least significant difference test (LSD), *p < 0.05, **p < 0.01)

Nissl staining was performed to compare the numbers of neurons in the hippocampi of the three groups (Figure 2A–C). In the sham group, hippocampal neuron soma were large, with dark cytoplasmic staining and lightly stained nuclei (Figure 2A). Quantification of neurons in different subregions of the hippocampus revealed that rats in the SD group had fewer neurons than those in the Sham group. There were more neurons in the CA1 and CA2 regions in the EA + SD group than in the SD group (Figure 2C). Thus, SD‐induced neuronal damage and loss of hippocampal neurons was rescued by EA.

Neurogenesis occurs continuously in the adult hippocampus, especially in the DG. To determine whether EA influenced hippocampal neurogenesis, proliferating cells were labeled with BrdU (Figure 2D). There were fewer DCX^+^ newborn neurons in the SD group compared with the sham group (Figure 2E). EA increased the number of DCX^+^ cells and percentage of BrdU^+^DCX^+^ neurons compared with the SD group (Figure 2E,F), while no difference was observed in the percentage of BrdU^+^NeuN^+^ mature neurons relative to the SD group (Figure S2A–C). There was no difference in brain weight among three groups (Figure S2D). These results show that EA prevents neuronal loss and promotes neurogenesis under SD.

### 3.3 EA increases the expression of synaptic proteins and preserves synapse structure under SD

**FIGURE 3:**
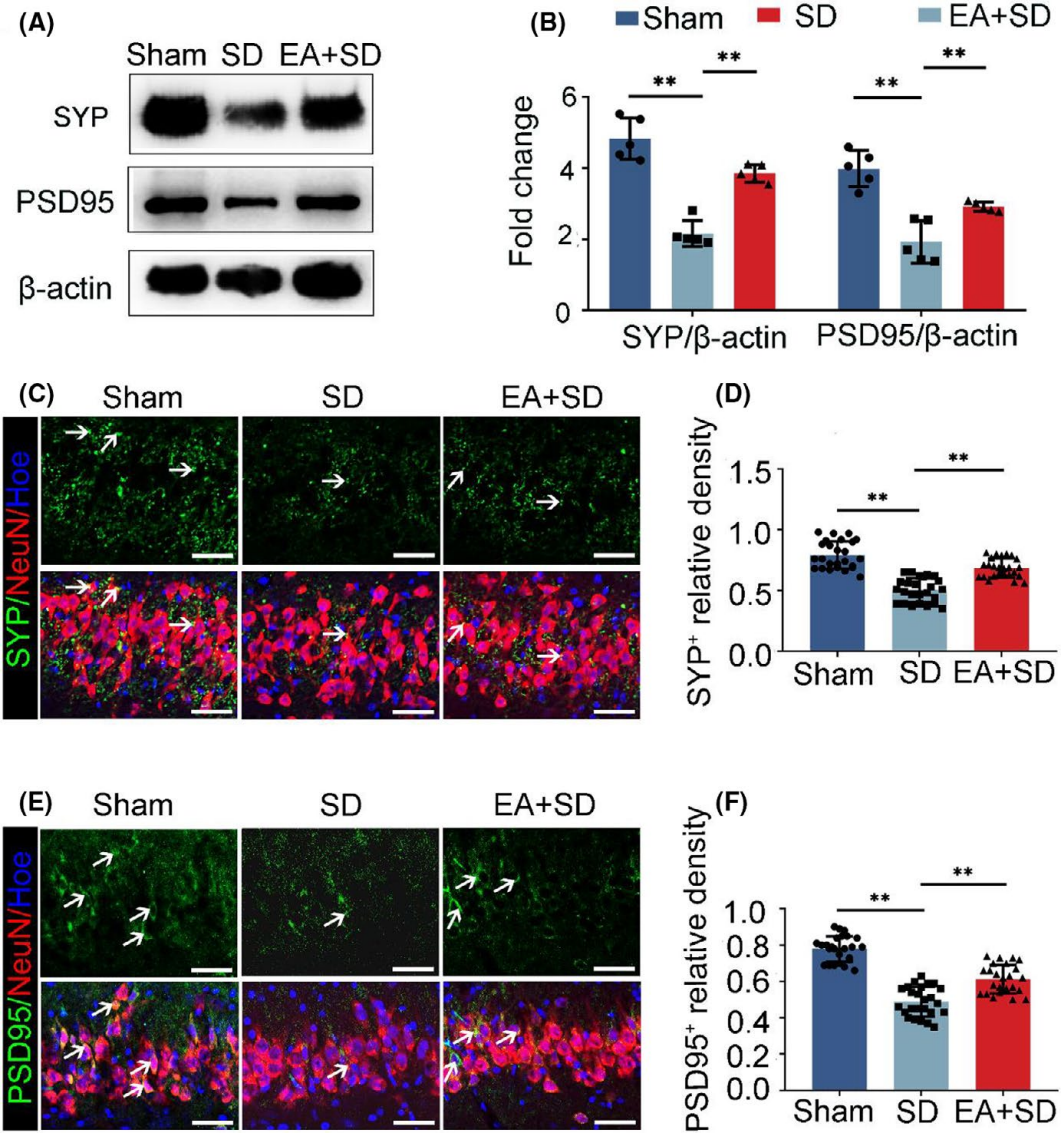
EA increases the expression of synaptic proteins in the hippocampus. (A) Western blot analysis of SYP and PSD95 levels in the hippocampus, with β‐actin as the loading control. (B) Quantification of SYP and PSD95 levels from the immunoblot experiment in panel A.( *n* = 5/group, data were presented as the mean ± SEM and analyzed by least significant difference test (LSD), *p < 0.05, **p < 0.01). (C) Colocalization of SYP (green) and the neuron marker NeuN (red) in the hilus of the hippocampus; nuclei were labeled with Hoechst (blue). Scale bar, 50 µm. (D) Relative density of SYP in the hilus of the hippocampus. (*n* = 5/group, data were presented as the mean ± SEM and analyzed by least significant difference test (LSD), *p < 0.05, **p < 0.01). (E) Colocalization of PSD95 (green) and NeuN (red) in the hilus; nuclei were labeled with Hoechst (blue). Scale bar, 50 µm. (F) Relative expression of PSD95 in the hilus of the hippocampus. (*n* = 5/group, data were presented as the mean ± SEM and analyzed by least significant difference test (LSD), *p < 0.05, **p < 0.01)

To determine whether EA affects neuronal synapses, we measured expression of the presynaptic marker synaptophysin (SYP) and the postsynaptic marker postsynaptic density (PSD)95 by WB and immunofluorescence analysis. SYP and PSD95 levels in the hippocampus were lower in the SD group than in the sham group but were increased in the EA + SD group (Figure 3A,B). SYP/NeuN and PSD95/NeuN double immunofluorescence labeling showed that hippocampal neurons in the hilus of the EA + SD group had higher SYP and PSD95 expression than those in the SD group (Figure 3C,E). Western blotting and densitometric analysis of SYP^+^ and PSD95^+^ levels in the hilus region confirmed this observation (Figure 3D,F). In contrast, the relative expression levels of SYP^+^ and PSD95^+^ in the CA1 and CA3 regions did not differ between the EA + SD and SD groups (Figure S3).

**FIGURE 4:**
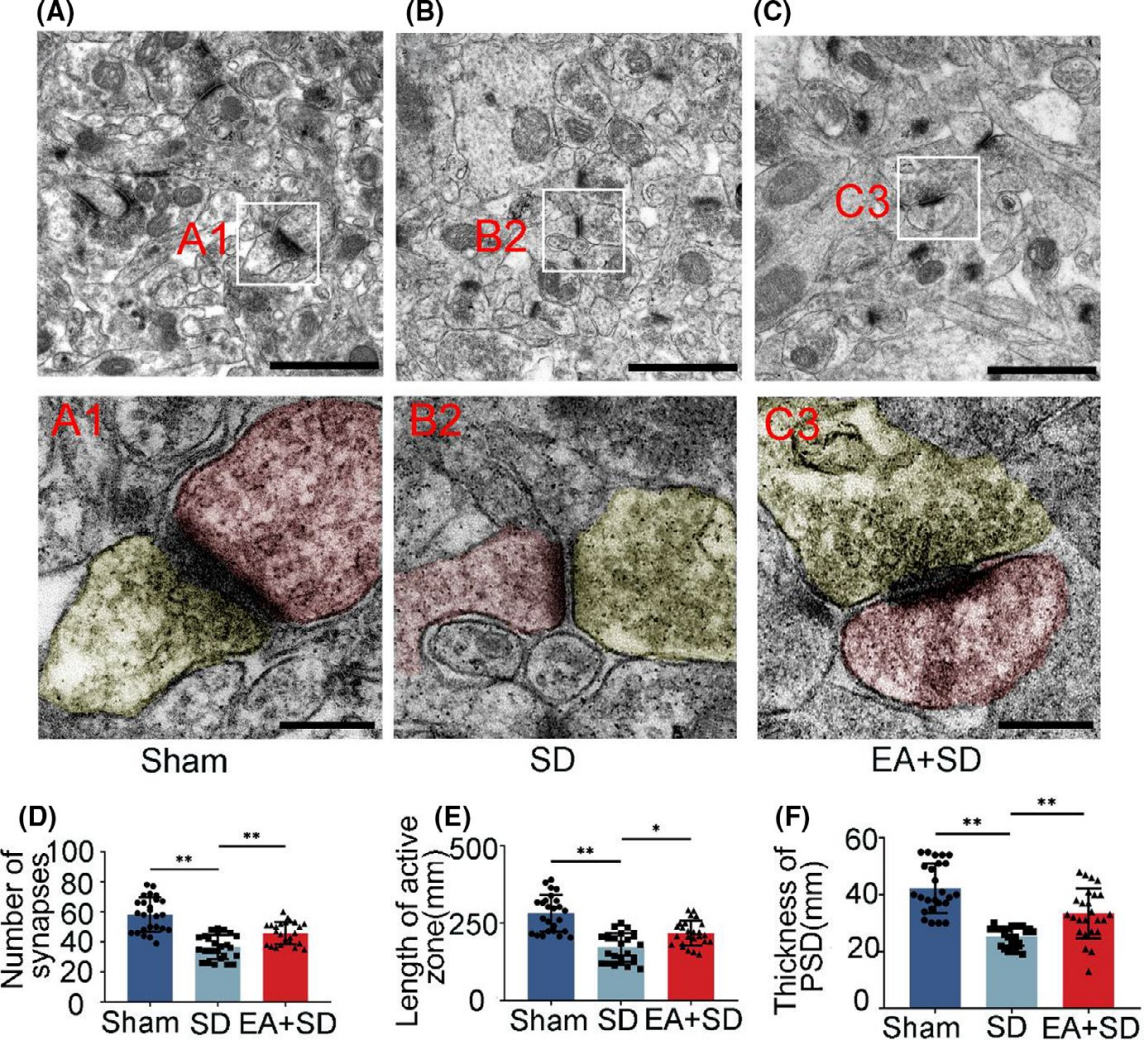
EA prevents and alleviates damage to synapse structure under SD. (A) TEM images of synapses in the hippocampus. Scale bar, 10 µm in panels A–C; 200 nm in panels A1–C1. Boxed areas show higher magnification views of presynaptic (yellow) and postsynaptic (red) terminals. (D–E) Quantitative analysis of synapse number (D), length of active zone (E), and PSD thickness (F) (*n* = 5/group, data were presented as the mean ± SEM and analyzed by least significant difference test (LSD) or nonparametric test (Kruskal‐Wallis test) (D: LSD test, E: Kruskal‐Wallis test, F: LSD test),*p < 0.05, **p < 0.01)

We examined the effects of SD and EA on hippocampal synapse structure by TEM. In the sham group, a typical synapse had distinct pre‐ and postsynaptic terminals separated by a synaptic cleft. Many synaptic vesicles were observed in the presynaptic terminal, while the postsynaptic terminal had a thicker membrane with a centralized PSD (Figure 4A–C and Figure S4A). Compared with the SD group, EA significantly increased synapse number, PSD thickness, and the length of the presynaptic active zone (Figure 4D–F), at the same time reduced the size of the synaptic cleft and curvature of the synaptic interface (Figure S4B,C). These results indicate that SD causes structural damage to hippocampal neuron synapses that are reversed by EA.

### 3.4 EA enhances BDNF/TrkB/Erk signaling under SD

**FIGURE 5:**
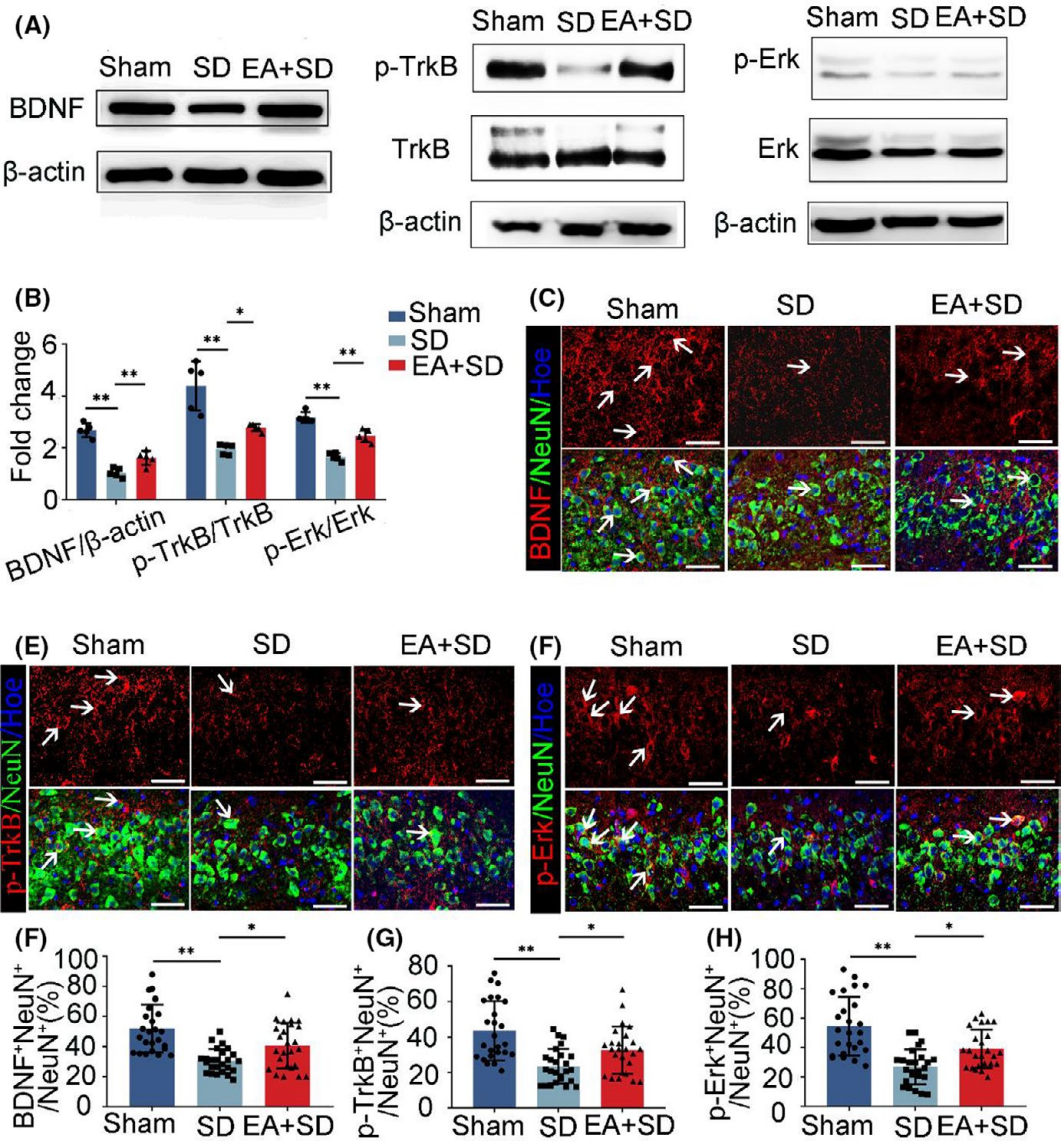
EA enhances BDNF/TrkB/Erk signaling in the hippocampus under SD. (A) Western blot analysis of BDNF, TrkB, p‐TrkB, ERK, and p‐ERK levels in the hippocampus, with β‐actin as the loading control. (B) Quantification of BDNF/β‐actin, p‐TrkB/TrkB, and p‐ERK/ERK levels from the immunoblot experiment in panel A. (*n* = 5/group, data were presented as the mean ± SEM and analyzed by one‐way ANOVA, *p < 0.05, **p < 0.01). (C–D) Colocalization of BDNF (red) and NeuN (green), p‐TrkB (red) and NeuN (green), and p‐ERK (red) and NeuN (green) in the DG of the hippocampus; nuclei were labeled with Hoechst (blue). Scale bar, 50 µm. (F–H) Percentages of BDNF + NeuN + /NeuN+,p‐TrkB + NeuN + /NeuN, and p‐ERK + NeuN + /NeuN + neurons. (*n* = 5/group, data were presented as the mean ± SEM and analyzed by nonparametric test (Kruskal‐Wallis test), *p < 0.05, **p < 0.01)

To investigate the molecular mechanism by which EA mitigates the effects of SD, we examined the expression levels of proteins in the BDNF/TrkB/Erk pathway in hippocampal neurons by ELISA and WB. The results demonstrated that EA significantly increased BDNF protein levels in the hippocampus (Figure 5A, Figure S5C). EA also enhanced TrkB and Erk phosphorylation compared with the SD group (Figure 5A,B). Immunofluorescence analysis revealed that BDNF and p‐TrkB were mostly localized in the cell membranes and soma, whereas p‐Erk was mainly present in the soma and nuclei (Figure 5C–E, Figure S5A,B). Moreover, the percentages of BDNF^+^NeuN^+^, p‐TrkB^+^NeuN^+^, and p‐Erk^+^NeuN^+^ cells in NeuN^+^ neurons were higher in the EA + SD group than in the SD group (Figure 5F–H). These results suggest that EA increases BDNF protein expression to activate the phosphorylation of TrkB receptors on the cell membranes of hippocampal neurons, leading to the phosphorylation of a downstream signaling molecule (Erk) of the TrkB receptor during SD.

### 3.5 Exogenous BDNF enhances synaptic protein expression and mEPSC frequency by inducing TrkB and Erk phosphorylation in hippocampal neurons

To assess whether EA‐increased BDNF promotes the survival and synaptogenesis of hippocampal neurons via TrkB/Erk signaling, cultured hippocampal neurons were treated with exogenous BDNF, then the expression of synaptic proteins (SYP and PSD95), mEPSCs, and TrkB and Erk phosphorylation were evaluated by immunofluorescence, whole‐cell patch clamp, and Western blot analyses.

**FIGURE 6:**
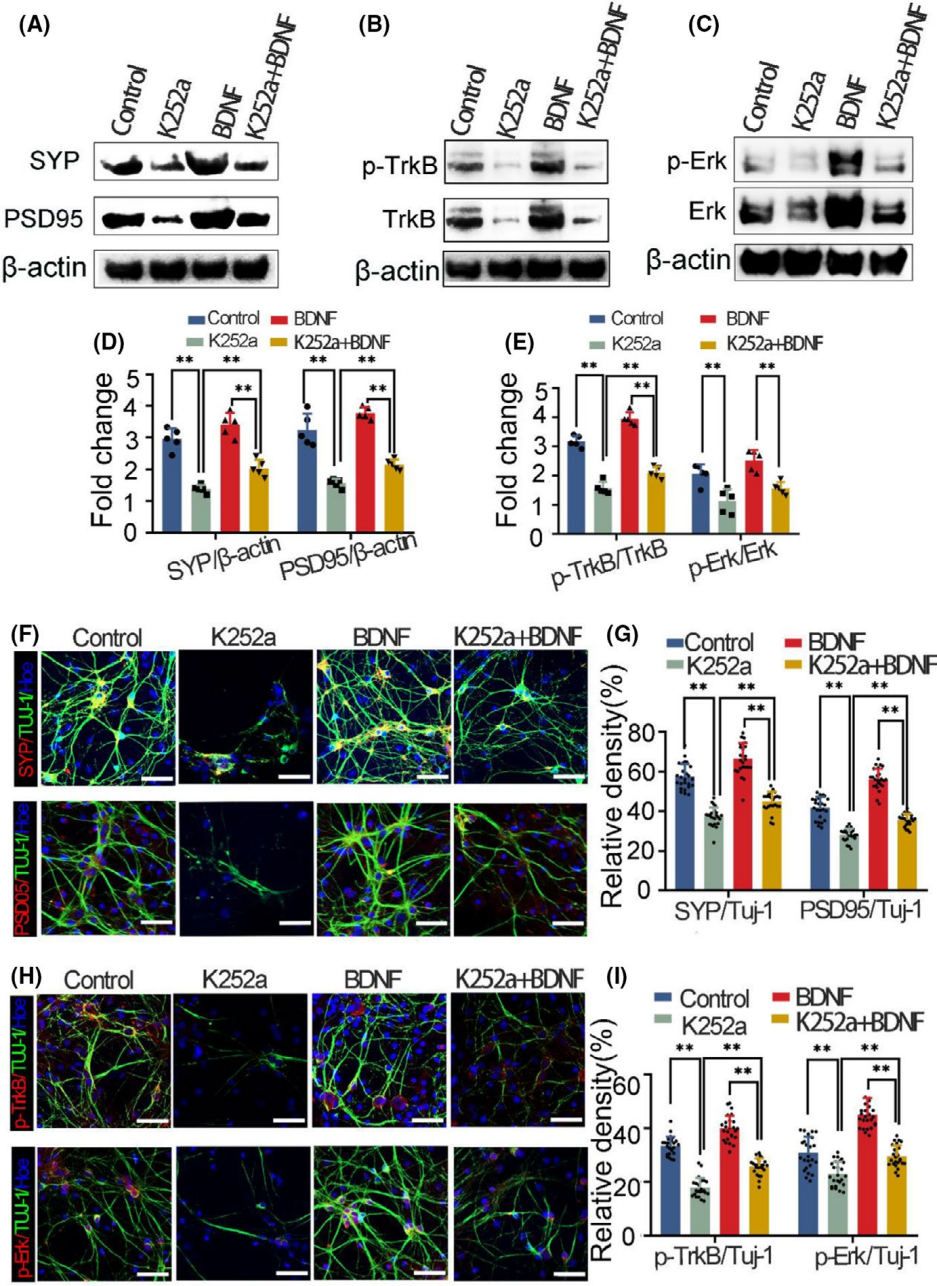
Exogenous BDNF enhances the expression of synaptic proteins and TrkB and ERK phosphorylation in primary hippocampal neurons. (A–C) Western blot analysis of SYP, PSD95, TrkB, p‐TrkB, Erk, and p‐Erk levels, with β‐actin as the loading control. (D) Quantification of SYP and PSD95 levels from the immunoblot experiment in panels A–C. (E) Western blot analysis of p‐TrkB/TrkB and p‐Erk/Erk levels. (*n* = 5/group, data were presented as the mean ± SEM and analyzed by least significant difference test (LSD), *p < 0.05). (F, H) Colocalization of SYP and PSD95 (red) with Tuj‐1 (green) (F) and of p‐TrkB and p‐Erk (red) with Tuj‐1 (green) (H); nuclei were labeled with Hoechst (blue). Scale bar 50 µm. (G, I) Relative densities of SYP/Tuj‐1 and PSD95/Tuj‐1 (G) and relative densities of p‐TrkB/Tuj‐1 and p‐Erk/Tuj‐1(I) (*n* = 5/group, data were presented as the mean ± SEM and analyzed by least significant difference test (LSD) or nonparametric test (Kruskal‐Wallis test) (SYP/Tuj‐1:LSD test, PSD95/Tuj‐1: Kruskal‐Wallis test, p‐TrkB/Tuj‐1: LSD test, p‐Erk/Tuj‐1: Kruskal‐Wallis test), *p < 0.05, **p < 0.01)

Compared with the control group, neuronal survival and synaptic expression (SYP and PSD95) were decreased in the presence of K252a, an inhibitor of the BDNF receptor TrkB, and application of exogenous BDNF reversed these effects (Figure 6A,D,F,G and Figure S6A,B). Furthermore, p‐TrkB and p‐Erk immunoreactivity in neurons was decreased in the K252a group compared to the control group, while BDNF increased p‐TrkB and p‐Erk expression in hippocampal neurons (Figure 6B,C,E,H, I, and Figure S6C). These results suggest that BDNF induced by EA promotes synapse formation by activating the downstream effectors TrkB and Erk. We also measured BDNF levels in the supernatant of hippocampal neuron cultures by ELISA after removing the exogenous factors and found that BDNF treatment induced BDNF production by the neurons and that the effect was blocked by K252a (Figure S6D). Under a holding potential of −70 mV, mEPSCs were detected. The results showed that the frequency of mEPSCs in the BDNF group was significantly higher than that in the K252a and K252a +BDNF groups (Figure S6E,F). Taken together, these findings suggest that exogenous BDNF enhances synaptic protein expression and synaptic transmission by inducing TrkB and Erk phosphorylation in hippocampal neurons.

## 4 DISCUSSION

We previously reported that EA improved cognitive performance—especially spatial learning and memory—that was impaired by sleep loss. However, the underlying mechanism was unknown. Neuron production, synapse formation, increased synaptic transmission, and plasticity contribute to enhanced cognitive function. In this study, EA alleviated SD‐induced impairment of spatial memory by promoting the survival, neurogenesis, and synaptic plasticity of hippocampal neurons via activation of the BDNF/TrkB/ERK signaling pathway.

SD is known to undermine hippocampus‐dependent spatial learning and memory.^39^ EA can mitigate cognitive dysfunction by inducing the functional reconstruction of neurons.^40^, ^41^ In this study, the SD model was established by the MMPM, which limits rapid eye movement (REM) sleep. In these rats, EA treatment improved spatial learning and memory in the MWM test. These findings suggest that EA improves cognitive function under SD by increasing REM sleep time. However, it remains to be determined whether it also affects non‐REM sleep.

DG neurons in adults are born in the subgranular zone but migrate within the DG and integrate into the existing neuronal circuitry, which may contribute to spatial learning.^42^, ^43^, ^44^ The effect of SD on neurogenesis in the DG has been investigated in various models.^7^, ^45^ EA reversed the SD‐induced decrease in neurogenesis, affecting both proliferation and neuronal differentiation. Nissl staining revealed that SD‐induced neuron damage was prevented by EA, particularly in the CA1 and CA2 regions of the hippocampus. Our finding that EA pretreatment promotes neuron survival and neurogenesis under SD is in line with previous reports.^46^, ^47^

Sleep consolidates recently learned information by facilitating the strengthening of synaptic connections. SD is associated with altered synapse structure and gene/protein expression,^48^, ^49^, ^50^ and acute SD has been shown to cause changes in synaptic plasticity in different hippocampal regions including the DG and CA1 area.^9^, ^51^, ^52^ In our study, EA reversed the SD‐induced downregulation of SYP and PSD95 proteins in the hippocampus, specifically in DG neurons. TEM analysis of synapse ultrastructure showed that EA prevented synapse loss and changes in synapse structure in the hippocampal neurons of the SD rats.

BDNF and its receptor TrkB are involved in the survival, neurogenesis, and synaptic plasticity of DG neurons.^53^, ^54^, ^55^ Binding of BDNF to TrkB activates various intracellular pathways including MAPK/Erk signaling.^56^, ^57^, ^58^ We found that EA promoted BDNF expression and TrkB phosphorylation on the cell membrane, resulting in the phosphorylation and activation of Erk in the nuclei of cultured hippocampal neurons. In vitro experiments showed that exogenous BDNF enhanced the expression of synaptic proteins and postsynaptic currents (mEPSCs) by promoting TrkB and Erk phosphorylation. The results suggested that exogenous BDNF promoted synaptic vesicle synthesis and release, as well as synaptic transmission in cultured hippocampal neurons. It was previously reported that BDNF is also released by microglia,^59^, ^60^ and whether this is involved in the protective effects of EA under SD warrants further study.

## 5 CONCLUSIONS

EA alleviated spatial memory impairment, prevented the loss of hippocampal neurons and synapses, promoted hippocampal neurogenesis, and enhanced the expression of BDNF, p‐TrkB, and p‐Erk in rats under SD. Notably, exogenous BDNF stimulated the expression of synaptic proteins and trans‐synaptic communication in the hippocampus. These results indicate that EA can mitigate the adverse effects of SD by modulating BDNF/TrkB/Erk signaling in the hippocampus, providing evidence for its therapeutic utility.

## CONFLICT OF INTEREST

The authors declare that they have no known competing financial interests or personal relationships that could have appeared to influence the work reported in this paper.

## AUTHOR CONTRIBUTIONS

Ying Ding and Jingwen Ruan conceived and designed the study and revised the manuscript. Wenya Pei, Qingwen Deng, Fanqi Meng, Baobao Zhang, and Yuan Gu developed the experimental methodology; performed the experiments; and wrote, reviewed, and revised the manuscript. Wenya Pei, Boyu Jiao, Jiuqing Tan, Haoyu Xu, Zhiling Li, Xin Zhou, and Guanheng He acquired, analyzed, and interpreted the data and performed statistical analyses. Ying Ding and Jingwen Ruan provided technical and material support. All authors read and approved the final paper.

## Supporting information

Appendix S1

Appendix S2

## Data Availability

The data that support the findings of this study are available from the corresponding author upon reasonable request.
