# Supplementary material for: Electroacupuncture promotes the survival and synaptic plasticity of hippocampal neurons and improvement of sleep deprivation‐induced spatial memory impairment
Source: CNS Neurosci Ther. 2021 Oct 8;27(12):1472–82. doi: 10.1111/cns.13722 (PMC8611786; doi:10.1111/cns.13722)
Supplement: Supplementary file 1 — Appendix S1 [file CNS-27-1472-s002.doc]

# Table S1 Primary and secondary antibodies

| Antibodies | Species | Type | Dilution | Source (Catalog) |
| --- | --- | --- | --- | --- |
| BrdU | Mouse | Monoclonal IgG | Dilution | Sigma-Aldrich,St.Louis,USA (SAB-4701040) |
| DCX | Rabbit | Polyclonal IgG | 1:500 | Abcam, London, UK (ab18723) |
| Synaptophysin (SYP) | Mouse | Monoclonal IgG | 1:500 | Abcam, London, UK (ab32127) |
| Postsynaptic Density Protein 95 (PSD95) | Mouse | Monoclonal IgG | 1:500 | Abcam, London, UK (ab13552) |
| Synaptophysin (SYP) | Rabbit | Monoclonal IgG | 1:1000 | Abcam, London, UK (ab8049) |
| Postsynaptic Density Protein 95 (PSD95) | Rabbit | Monoclonal IgG | 1:1000 | Abcam, London, UK (ab238135) |
| NeuN | Rabbit | Monoclonal IgG | 1:1000 | Abcam, London, UK (ab177487) |
| NeuN | Mouse | Monoclonal IgG | 1:300 | Abcam, London, UK (ab104224) |
| BDNF | Rabbit | Monoclonal IgG | 1:500 | Invitrogen, California,  USA (PA5-85730) |
| TrkB | Rabbit | Polyclonal IgG | 1:500  1:300 | Invitrogen, California,  USA (PA5-14903) |
| p-TrkB | Rabbit | Polyclonal IgG | 1:500 | Invitrogen, California,  USA (PA5-36695) |
| Erk | Rabbit | Polyclonal IgG | 1:500 | Invitrogen, California,  USA (61-7400) |
| p-Erk | Rabbit | Polyclonal IgG | 1:1000 | Invitrogen, California,  USA (36-8000) |
| β-ⅢTubulin（Tuj-1） | Mouse | Monoclonal IgG | 1:1000 | Abcam, London, UK (ab78078) |
| GFAP | Chicken | Monoclonal IgG | 1:1000 | Abcam, London, UK (ab124436) |
| Goat polyclonal Secondary Antibody to Rabbit IgG H&L (Alexa Fluor® 555) | Goat | Monoclonal IgG | 1:1000 | Abcam, London, UK (ab150113) |
| Goat polyclonal Secondary Antibody to Mouse IgG H&L (Alexa Fluor® 488) | Goat | Monoclonal IgG | 1:1000 | Abcam, London, UK (ab150113) |
| Goat Anti-Rabbit (HRP) | Goat | Polyclonal IgG | 1:3000 | Abcam, London, UK (ab150077) |


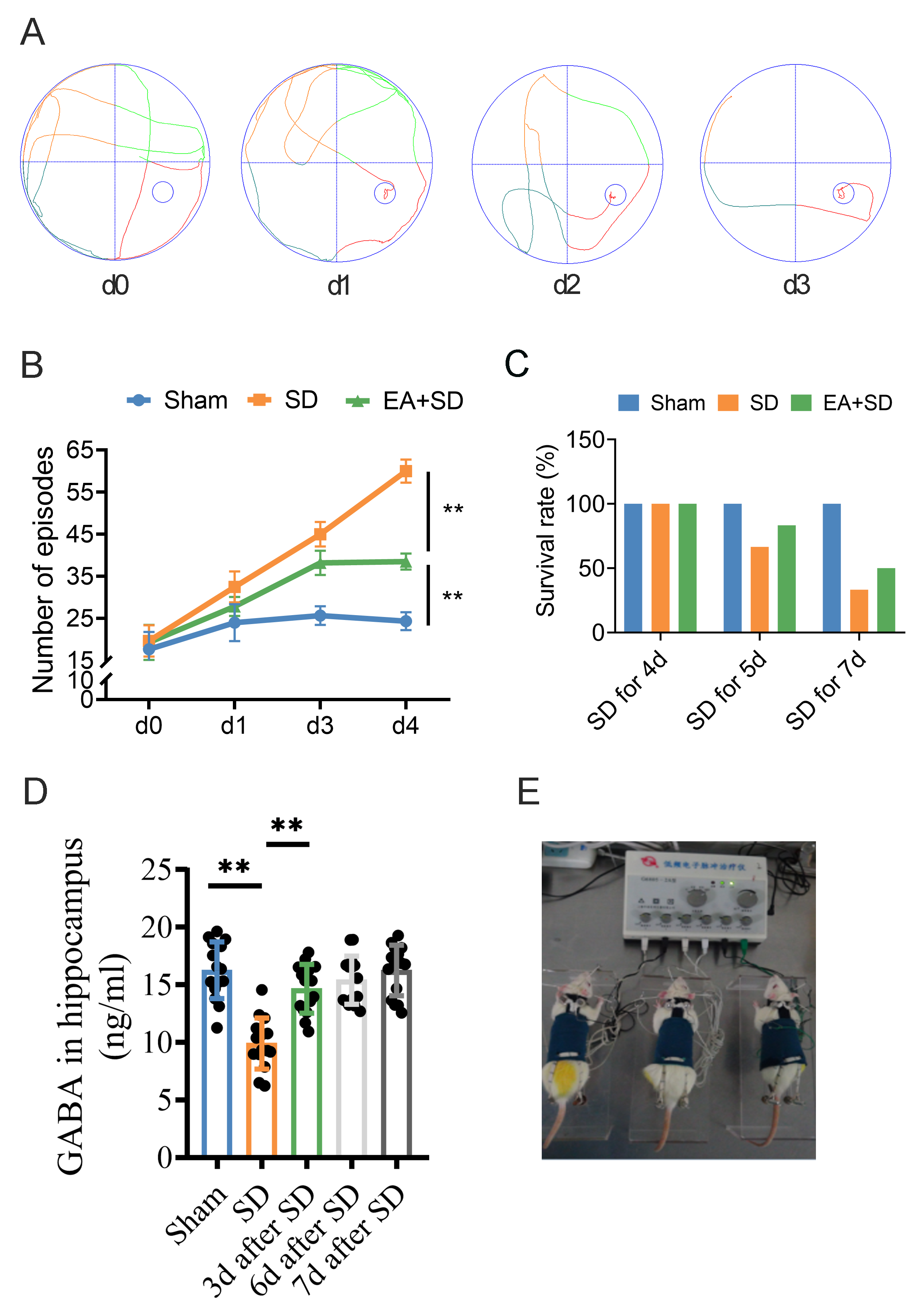


**Figure S1.** EA ameliorated spatial memory impairment induced by sleep deprivation. Related to Figure 1. (A)Trace plot in the training phase with a hidden platform in the Sham group. (B)The number of episodes in the training phase. (n=6/group, data were presented as the mean ± SEM and analyzed by least signiﬁcant difference test (LSD), *p < 0.05, **p < 0.01) (C) Survival rate of SD for different days. (D) GABA level in hippocampus. (Data were presented as the mean ± SEM and analyzed by nonparametric test (Kruskal-Wallis test), *p < 0.05, **p < 0.01). (E) The process of EA treatment.


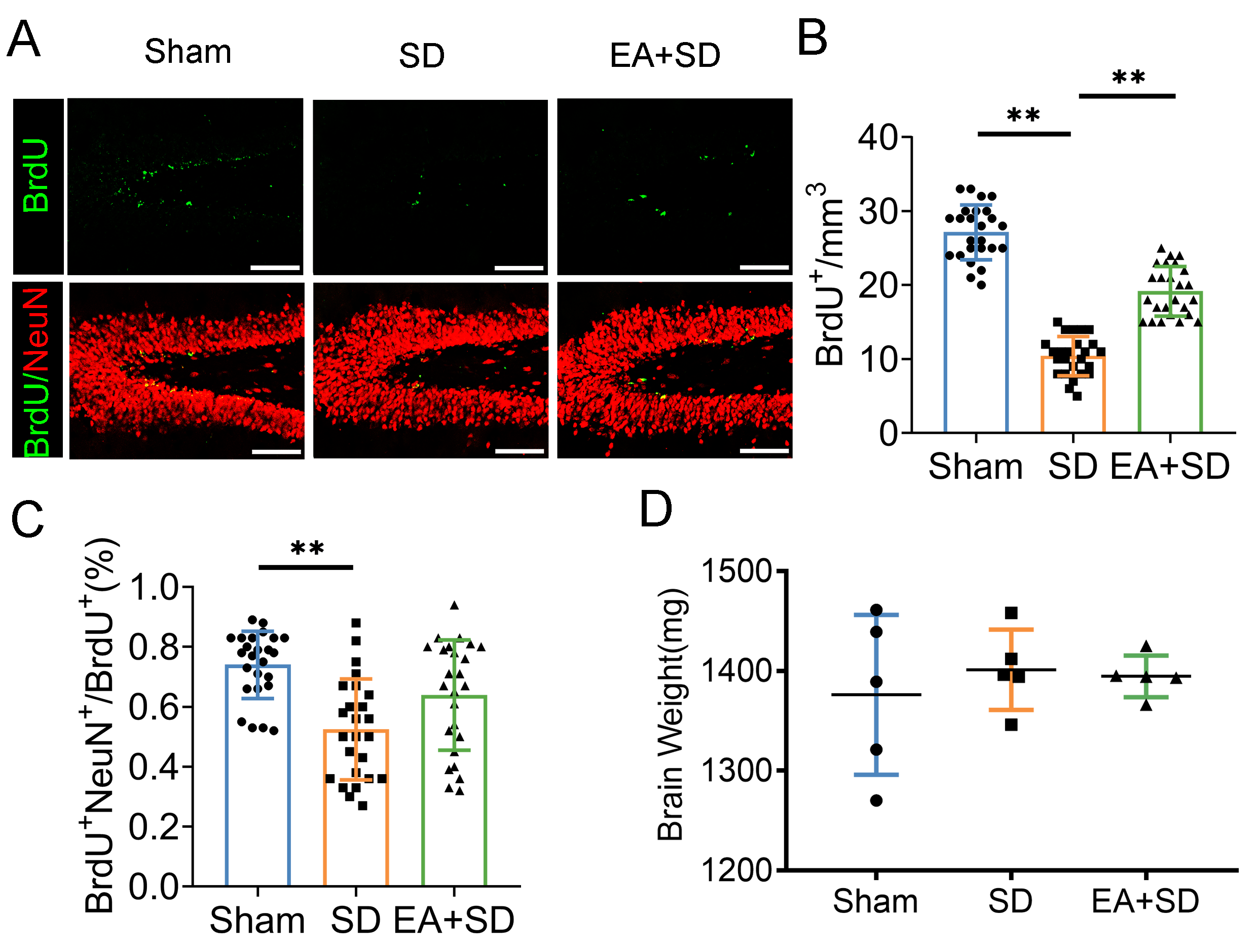


**Figure S2.** EA promote the neurogenesis reduced during SD. Related to Figure 2. (A)Representative images show co-localization of NeuN (red) and BrdU (green), (Scale bar=50 µm), the boxed areas are shown with their corresponding higher magnification images. (B)Bar chart of the percentage of BrdU^+^NeuN^+^ cells colocalized with total BrdU^+^. (n=5/group, data were presented as the mean ± SEM and analyzed by least signiﬁcant difference test (LSD), *p < 0.05, **p < 0.01) (C)Bar chart of the number of BrdU^+^ cells. (n=5/group, data were presented as the mean ± SEM and analyzed by nonparametric test (Kruskal-Wallis test), *p < 0.05, **p < 0.01) (D)Bar chart of brain weight.

**
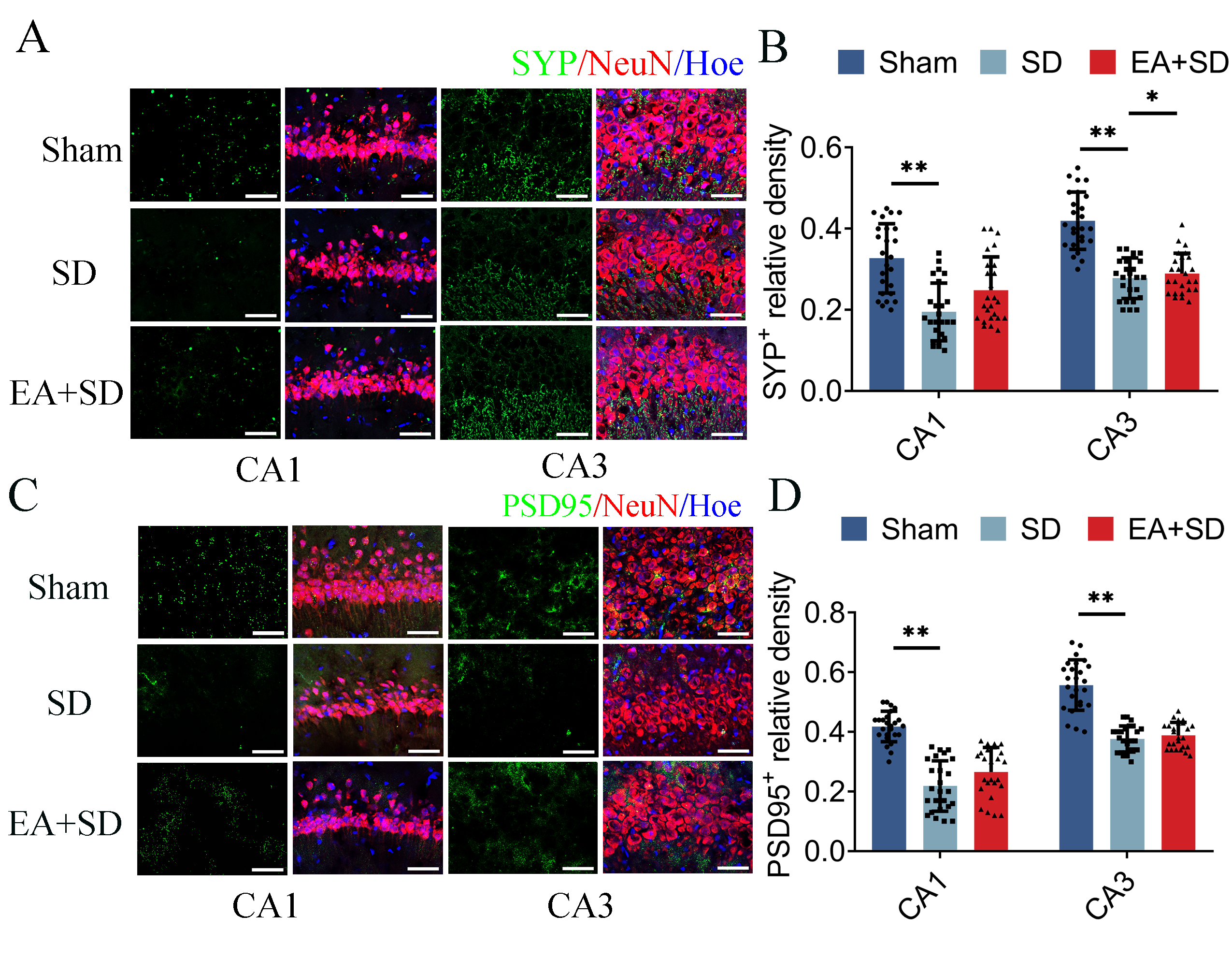
Figure S3.** The expression of synapse-associated protein SYP and PSD95 in regions of CA1 and CA3 in hippocampus. Related to Figure 3. (A)Representative images show co-localization of SYP (green) and NeuN (red) from CA1 and CA3, with Hoechst labeling of cell nuclei (blue) (Scale bar=50 µm). (B)Bar chart showing relative density of SYP in CA1, CA3 region of the hippocampus (n=5/group, data were presented as the mean ± SEM and analyzed by least signiﬁcant difference test (LSD) or nonparametric test (Kruskal-Wallis test)（CA1: LSD test, CA3: Kruskal-Wallis test）,*p < 0.05, **p < 0.01). (C)Representative images show co-localization of PSD95 (green) and NeuN (red) from CA1 and CA3, with Hoechst labeling of cell nuclei (blue) (Scale bar=50 µm). (D)Bar chart of relative density of PSD95 in CA1, CA3 region of the hippocampus. (n=5/group, data were presented as the mean ± SEM and analyzed by least signiﬁcant difference test (LSD) or nonparametric test (Kruskal-Wallis test)（CA1: LSD test, CA3: Kruskal-Wallis test）,*p < 0.05, **p < 0.01).


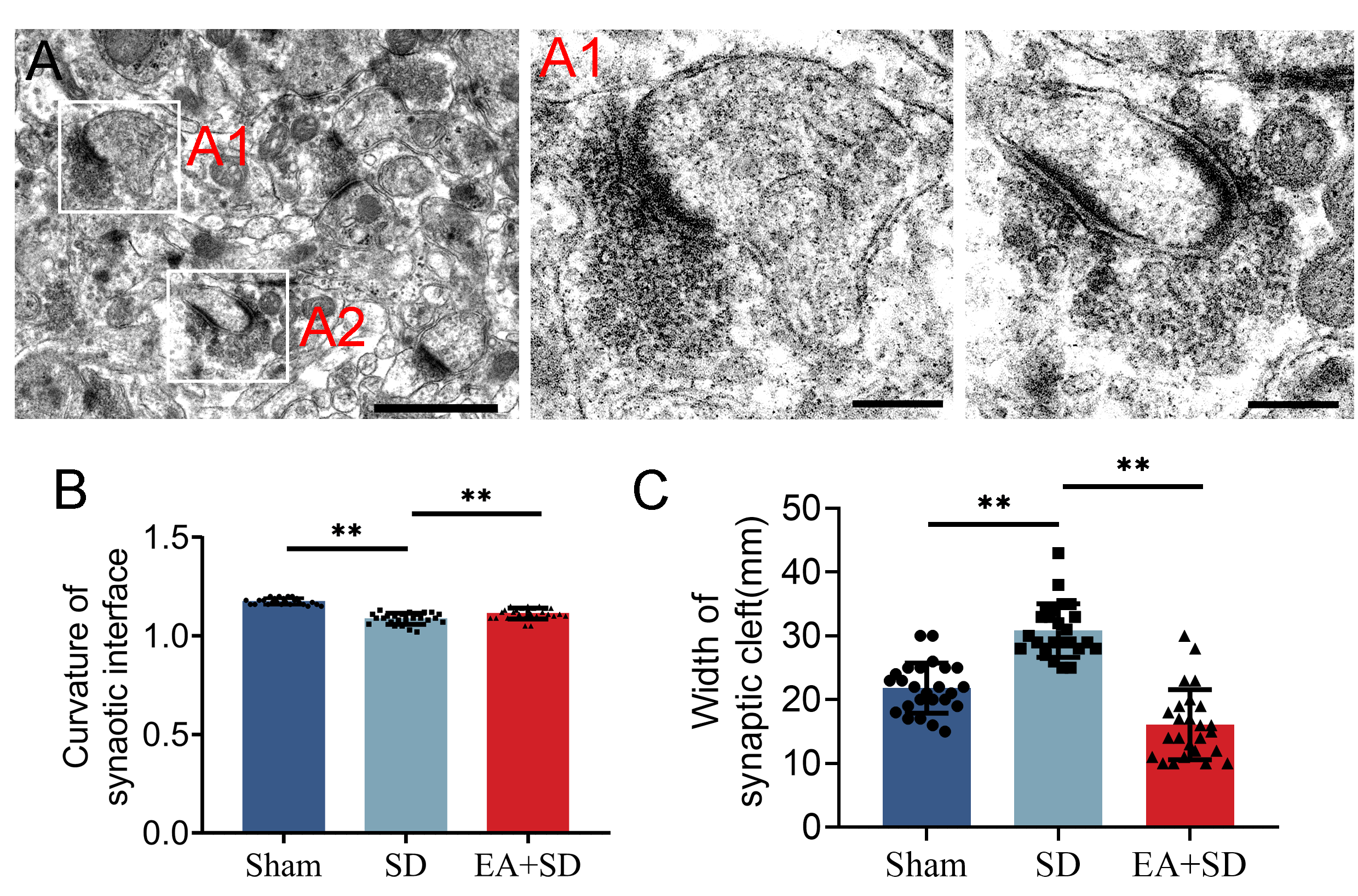


**Figure S4.** EA repaired the damage of synapse structure during SD. Related to Figure 4. (A)TEM images show the structure of synapses in the Sham group (Scale bar=10µm in (A); 200nm in (A1)(A2)), the boxed areas are shown with their corresponding higher magnification images. (B)Bar chart showing curvature of synaptic interface. (n=5/group, data were presented as the mean ± SEM and analyzed by least signiﬁcant difference test (LSD), *p < 0.05, **p < 0.01) (C)Bar chart showing width of synaptic cleft. (n=5/group, data were presented as the mean ± SEM and analyzed by nonparametric test (Kruskal-Wallis test), *p < 0.05, **p < 0.01).


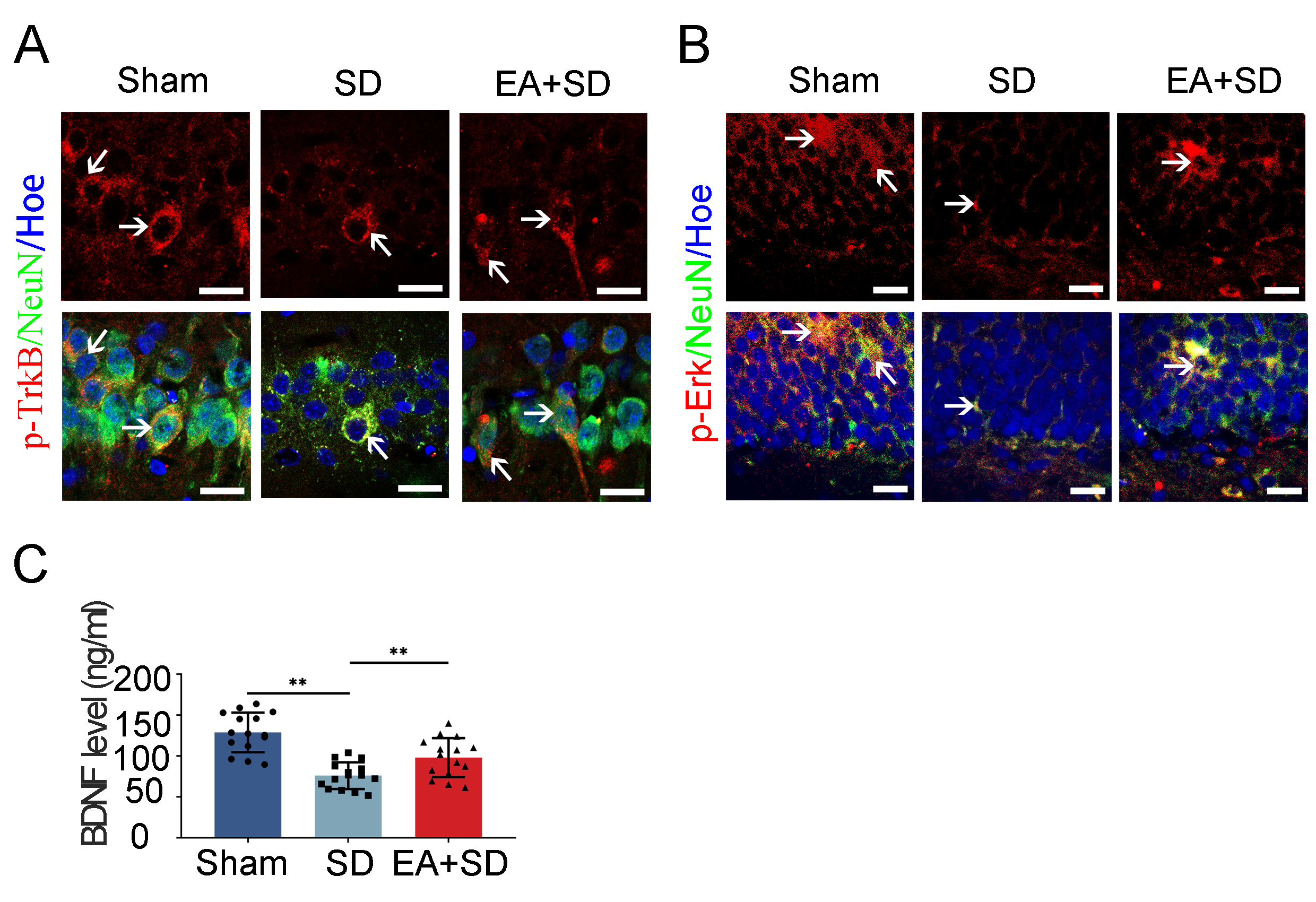


**Figure S5.** EA upregulated BDNF/TrkB/Erk signaling pathway during SD in hippocampus. Related to Figure 5. (A)Representative images show co-localization of p-TrkB (red) and NeuN (green) in the CA1, with Hoechst labeling of cell nuclei (blue) (Scale bar 20 µm). (B)Representative images show co-localization of p-Erk (red) and NeuN (green) in the DG, with Hoechst labeling of cell nuclei (blue) (Scale bar 20 µm). (C)BDNF level in the hippocampus (n=5/group, data were presented as the mean ± SEM and analyzed by one-way ANOVA, *p < 0.05, **p < 0.01).


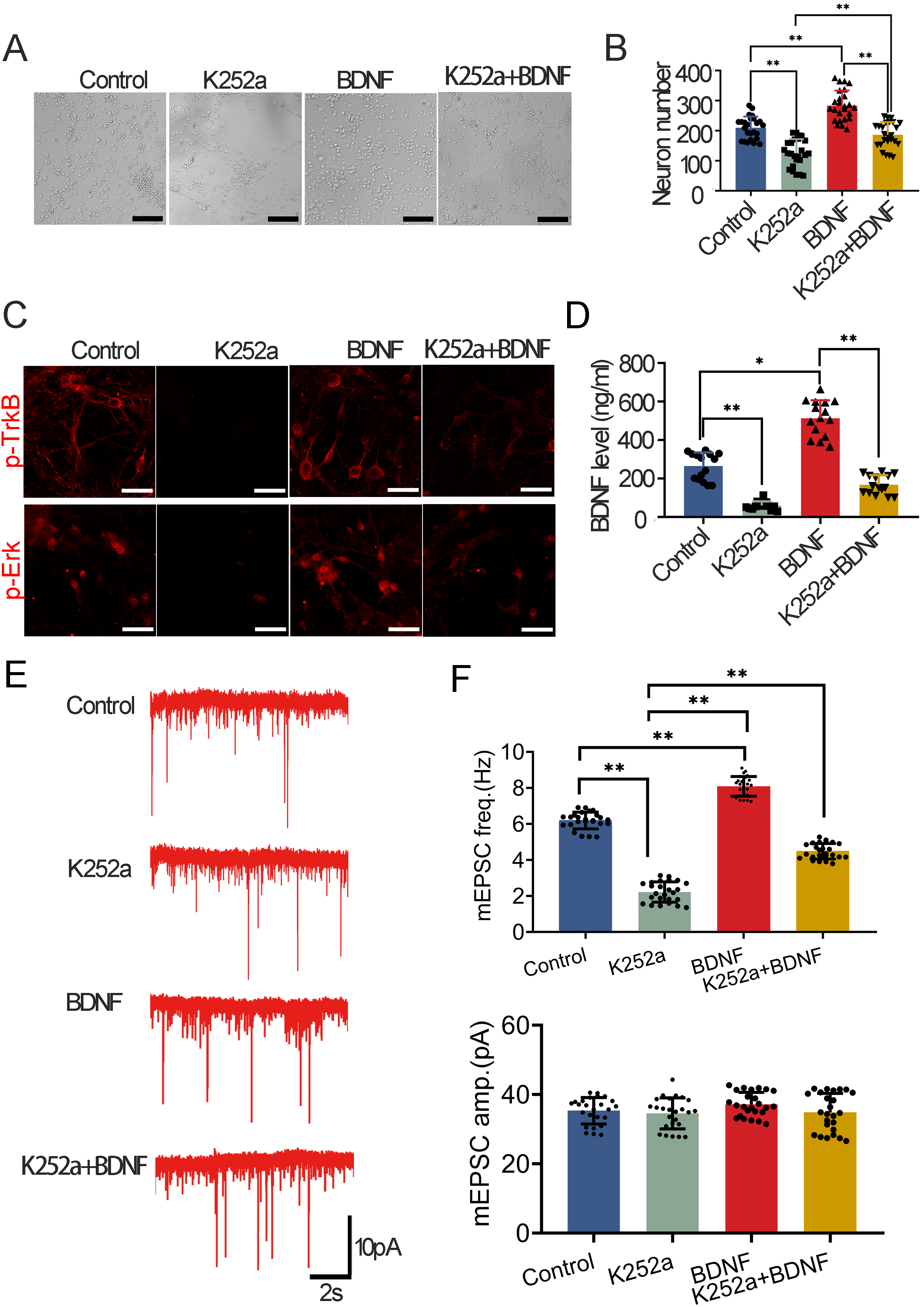


**Figure S6.** Exogenous BDNF promote neuron survival in primary hippocampal neurons. Related to Figure 6. (A)Representative images show neuron cells in light microscopy (Scale bar=100 µm). (B)Bar chart showing neuron numbers. (n=5/group, data were presented as the mean ± SEM and analyzed by least signiﬁcant difference test (LSD), *p < 0.05, **p < 0.01). (C)Representative images show p-TrkB (red) and p-Erk (red)(Scale bar 50 µm). (D)Bar chart of BDNF level in cell supernatant from primary culture of hippocampus neurons. (n=5/group, data were presented as the mean ± SEM and analyzed by nonparametric test (Kruskal-Wallis test), *p < 0.05, **p < 0.01). (E) Showing the representative images of mEPSCs in four groups. (F) Bar charts showing the frequency and amplitude of mEPSCs. (n=5/group, data were presented as the mean ± SEM and analyzed by nonparametric test (Kruskal-Wallis test), *p < 0.05, **p < 0.01).
