# Supplementary figures and images for: Electroacupuncture promotes the survival and synaptic plasticity of hippocampal neurons and improvement of sleep deprivation‐induced spatial memory impairment
Source: CNS Neurosci Ther. 2021 Oct 8;27(12):1472–82. doi: 10.1111/cns.13722 (PMC8611786; doi:10.1111/cns.13722)

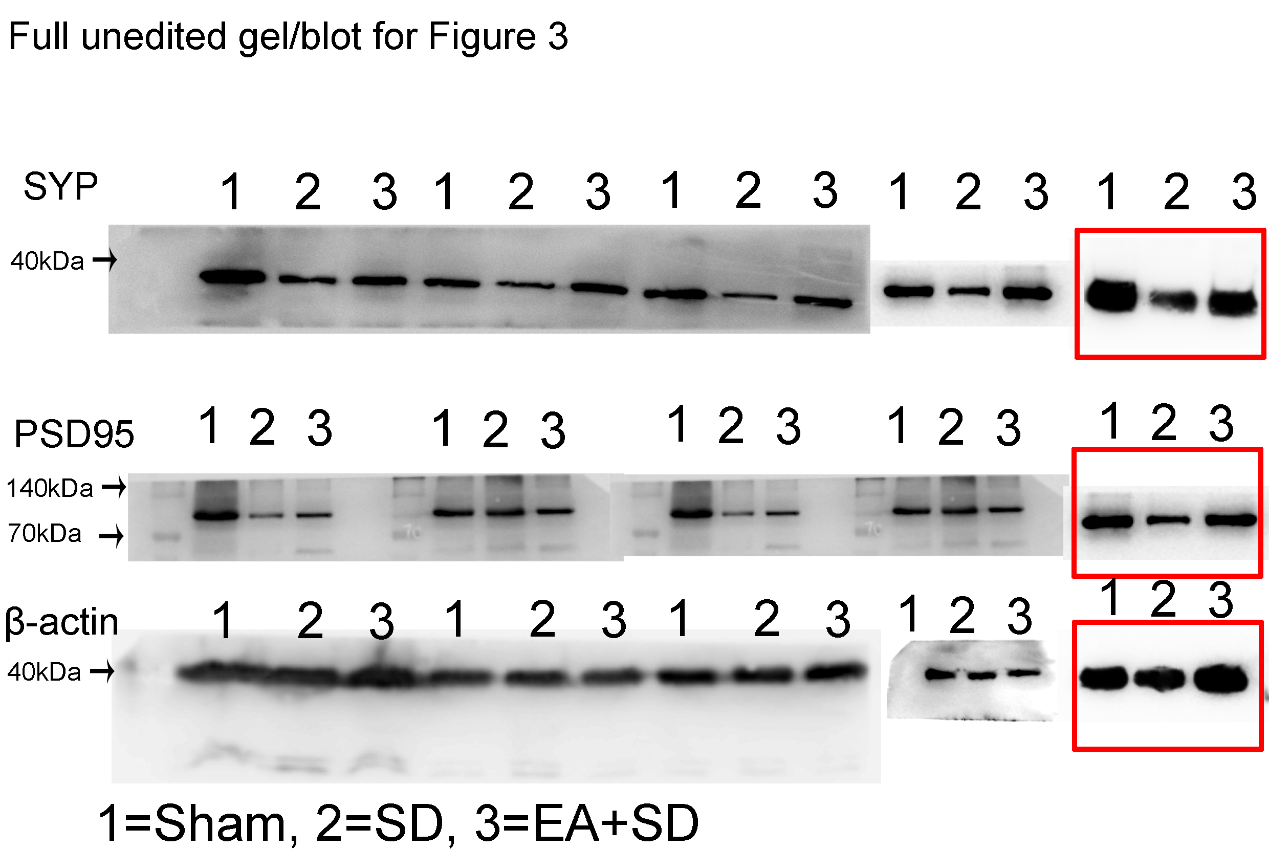


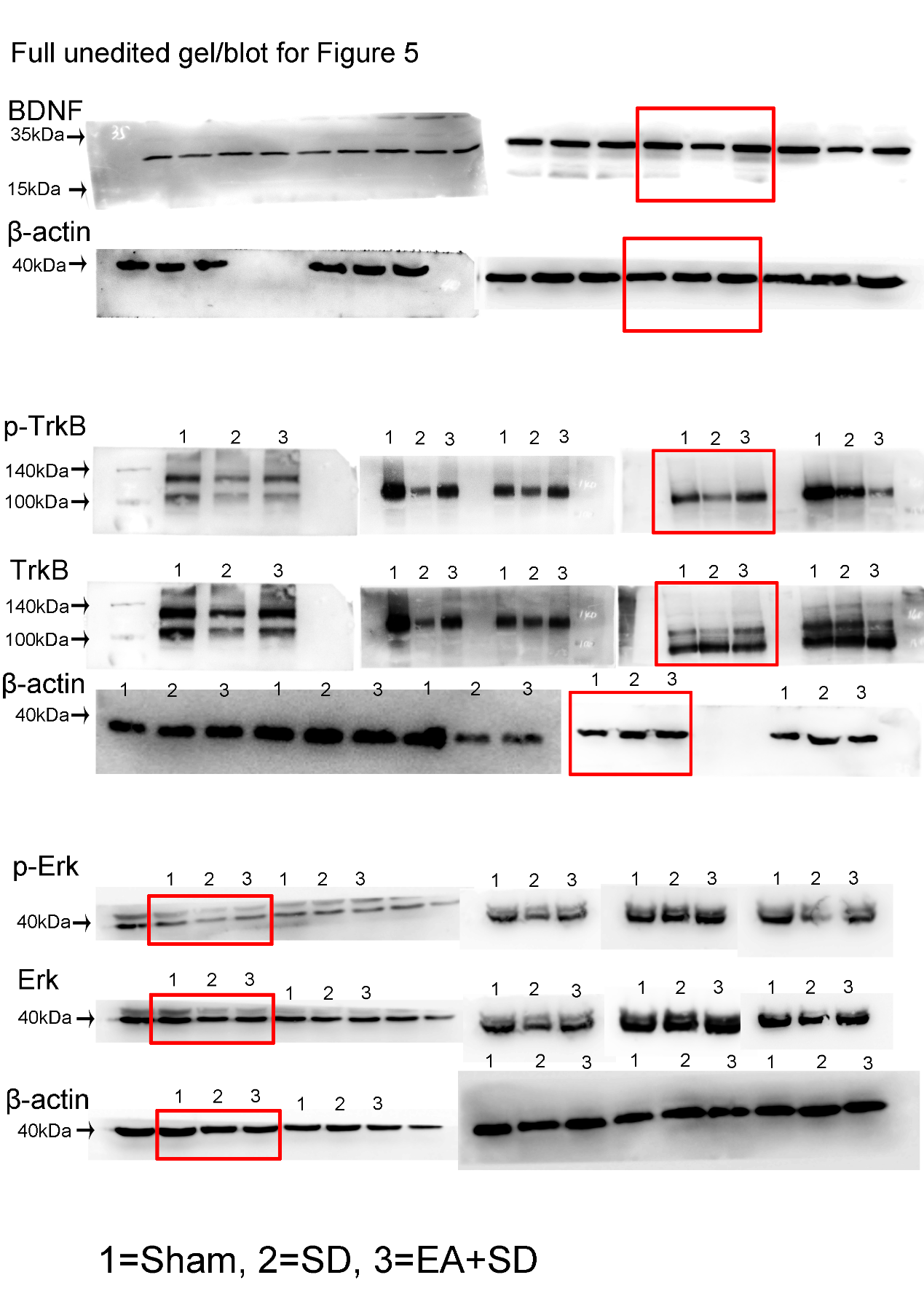


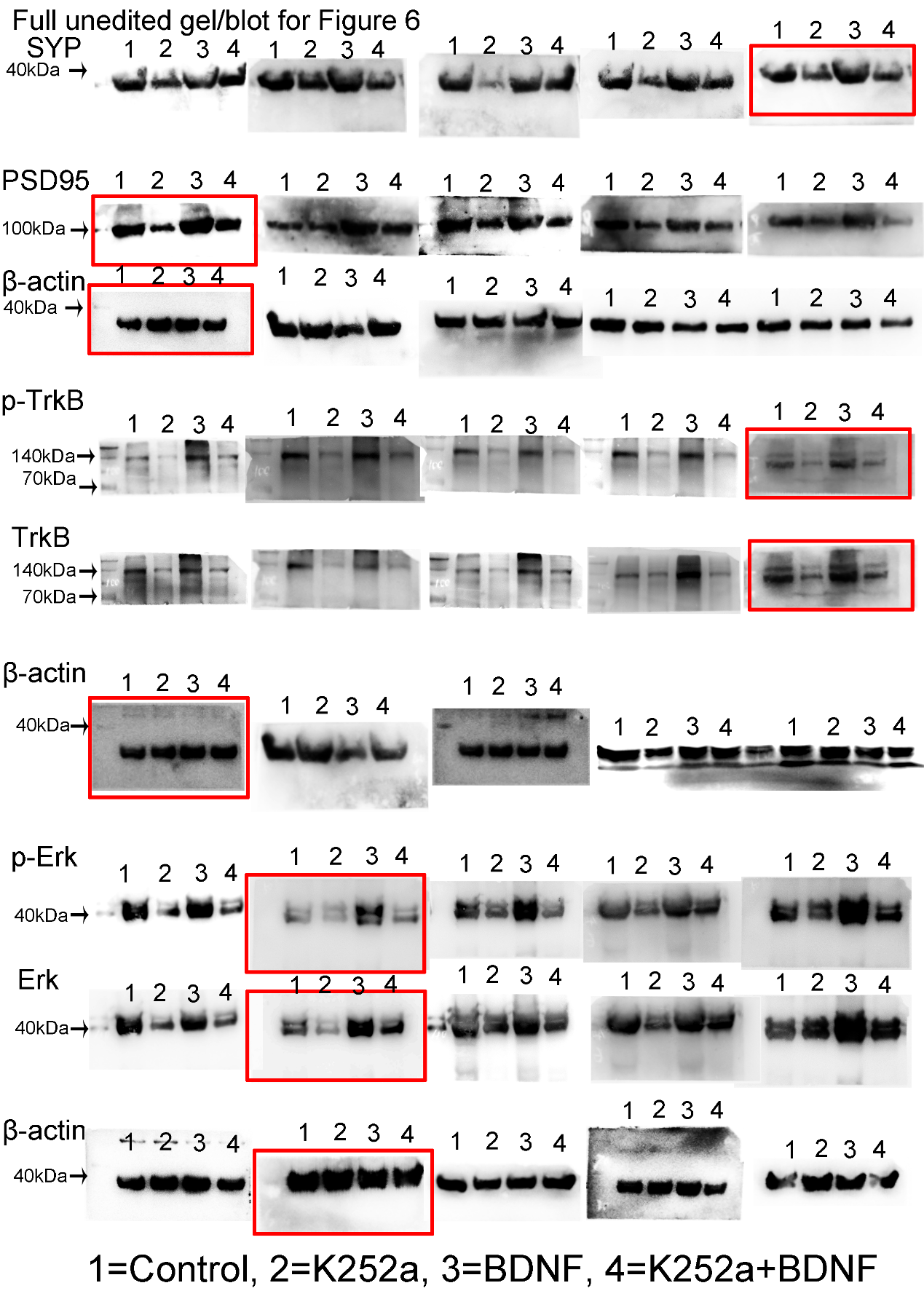

Supplement: Supplementary file 2 — Appendix S2 [file CNS-27-1472-s001.docx]
